# Deep application of controlled-release urea increases the yield and saponin content of *Panax notoginseng* by regulating soil nitrate distribution

**DOI:** 10.3389/fpls.2024.1505702

**Published:** 2025-01-23

**Authors:** Yun-xia Su, Ping Zhao, Li-jie Jia, Yuan-feng Cao, Guan-ze Liu, Jun-wen Chen, Sheng-chao Yang, Yan-li Zhou, Guang-qiang Long

**Affiliations:** ^1^ Yunnan Agricultural University, College of Environment and Resources, Kunming, China; ^2^ The Key Laboratory of Medicinal Plant Biology of Yunnan Province, Yunnan Agricultural University, Kunming, China; ^3^ Yunnan Agricultural University National and Local Joint Engineering Research Center on Germplasm Innovation & Utilization of Chinese Medicinal Materials in Southwest China, Kunming, China; ^4^ Germplasm Bank of Wild Species & Yunnan Key Laboratory of Crop Wild Relatives Omics, Kunming Institute of Botany, Chinese Academy of Sciences, Kunming, China

**Keywords:** deep application, yield, saponin content, nitrate nitrogen distribution, controlled-release urea

## Abstract

**Introduction:**

The deep application of controlled-release urea (CRU) offers potential advantages for crops with extended growth periods. However, its effects on *P. notoginseng* yield and quality, a medicinal plant with a prolonged nutrient acquisition duration, remain unclear.

**Methods:**

In this study, we conducted a two-year field plot experiment to investigate the effect of CRU on *P. notoginseng* with three placement depths (0, 6, and 12 cm denoted as R0, R6, and R12, respectively) at an application dosage of 250 kg N ha^-1^ with biochar addition (R6B) and 20% N reduction (R6R) based on the R6, with conventional fertilization (250 kg N ha^-1^, common urea) serving as the control (CK).

**Results:**

Our results indicated that yields increased by 27.1–37.6% with R0, R6, R12, and R6B, while remaining stable with R6R compared to CK. Simultaneously, the total saponin content in the roots of R6, R6B, and R6R was improved by 14.3–38.1%, compared to CK. The distribution depth of soil NO_3_^⁻^-N and plant roots increased with the depth of CRU application, with a high overlap in time and space, indicating *P. notoginseng* N uptake peaked when CRU was applied at a depth of 6 cm (R6). Structural equation modeling indicated that soil NO_3_^⁻^-N supply in specific microareas directly affected the N uptake and increased total saponin content by increasing root length and surface area, thus boosting yield.

**Conclusion:**

This study identifies that the deep application of CRU at a depth of 6 cm has the potential to enhance both yield and quality of *P. notoginseng* and highlights that the spatial-temporal matching of soil NO₃⁻-N and plant roots was the key to applying CRU to ensure high yield and quality.

## 1 Introduction

Controlled-release urea (CRU), typically coated with hydrophobic substances (Xu et al., 2022), provides a gradual and manageable release of nitrogen (N). A single application of CRU can meet the N nutrient demands of crops throughout their entire growth cycle, especially for crops with slow and continuous nutrient acquisition (Zhou et al., 2023). Compared to traditional polymer-based coatings, biodegradable polyurethane coatings offer significant advantages in environmental sustainability, improving nutrient utilization and effectively reducing negative environmental impacts (Patel et al., 2024; Yang et al., 2024). As costs and prices have gradually decreased, this CRU has been used in the production of grain crops, including corn and wheat (Zheng et al., 2016). CRU is generally applied to the root zones of crops to match the soil moisture and temperature required for appropriate nutrient release and to maximize the accessibility and effectiveness of released N (Grant et al., 2012). Consequently, the fertilization depth of CRU significantly affects crop N acquisition, quality, and the yield of agricultural crops (Zhang et al., 2023). As the depth of fertilization increases, soil moisture and soil nitrification weakens owing to decreased oxygen concentration and increased moisture, which prolongs the retention time of NH_4_^+^-N and narrows the spatial range of available N distribution (Rychel et al., 2020; Xia et al., 2021). In contrast, the deep application of fertilizers changes the root system characteristics, spatial allocation, and nutrient uptake (Mumtahina et al., 2023), which further regulates the N content and N transformation enzyme activity in plants (Wang et al., 2022). Furthermore, it was observed that plant N concentration affects secondary metabolic processes, thereby altering the accumulation of secondary metabolites (SMs) in plant tissues (Ahanger et al., 2019).

Currently, the deep application of CRU is primarily focused on fruit trees and grain crops, with most research examining its effects on N use efficiency and yield. The CRU application in the cultivation of traditional Chinese medicine, such as *Panax notoginseng* F. H. Chen (*P. notoginseng*), has not been reported yet (Wang et al., 2011). The roots of *P. notoginseng* have been extensively utilized to extract various medicinal active substances, including saponins and flavonoids, which can effectively treat cerebrovascular diseases (Li et al., 2020). Generally, after seedlings are transplanted to the field, *P. notoginseng* requires continuous field growth for two years, with an annual growth period exceeding seven months (Tang et al., 2021). During this long annual growth period, nutrients are continuously absorbed from the soil at a low intensity. Therefore, considering the compatibility between the nutrient release dynamics of CRU and the nutrient acquisition characteristics of *P. notoginseng*, it is essential to evaluate the applicability and potential applications of CRU in *P. notoginseng* fertilization.

In addition, CRU was also applied in reduced amounts or in combination with other fertilizer enhancers for further leveraging its advantages. Previous studies have shown that reducing N input maintained maize yield when CRU was applied (Hu et al., 2023), especially considering the excessive N application in current planting practices; and N fertilization with a relatively low level facilitated the enhancement of *P. notoginseng* quality via increasing the content of active components (Cun et al., 2024). Additionally, biochar exhibits nutrient retention characteristics and is expected to further immobilize NH_4_^+^-N, reduce N transport and losses, and thereby enhance the effectiveness of N fertilizers (Hou et al., 2022; Shang et al., 2023). Nonetheless, there is still a lack of research evidence on reducing N input or using biochar based on CRU application to prolong N accessibility, improve the productivity and quality of *P. notoginseng*.

To examine the performance of the deep application of CRU in terms of the productivity and quality of *P. notoginseng*, we conducted a two-year field experiment with varying application depths, biochar addition, and N reduction. We hypothesized the: (1) applying CRU at a depth of 6 cm can effectively enhance the yield and quality of *P.notoginseng*, and that reducing N by 20% based on CRU application at the same depth can maintain the yield of *P. notoginseng*. (2) Applying CRU at a depth of 6 cm with the addition of biochar can effectively meet the nutrient demands of *P. notoginseng* in its later growth stages, thereby improving its yield and quality. (3) The spatio-temporal distribution of NO_3_^−^-N content is a key determinant of N uptake, crop yield, and the concentration of active ingredients (i.e.,SMs) of *P.notoginseng*.

## 2 Materials and methods

### 2.1 Experimental site

The field research took place from 2022 to 2023 in Zehei town, Luquan County, Kunming, Southwest China (26°14′N, 102°69′E). The location is situated at an elevation of 2,588 m, experiencing a subtropical monsoonal climate. The average yearly temperature is 15.6°C, with annual precipitation ranging from 800 to 1,200 mm. The soil type is mountainous red soil. Before the commencement of the experiment, the surface soil (0-20 cm) exhibited the following properties: organic matter at 32.85 g kg^−1^, total N at 1.69 g kg^−1^, NO_3_^−^-N at 3.54 mg kg^−1^, available phosphorus (AP) at 41.69 mg kg^−1^, available potassium (AK) at 290.99 mg kg^−1^, and a pH value of 6.37.

### 2.2 Experimental design

This study employed a randomized block design, encompassing six distinct treatments. These included conventional fertilization serving as the control (CK), CRU application at three depths (0, 6, and 12 cm, denoted as R0, R6, and R12, respectively), biochar addition (R6B), and 20% N reduction (R6R) based on the R6 treatment (**Table 1**). Each treatment was replicated three times within a 20 m² plot (4 m × 5 m).

**Table 1:** Nitrogen fertilizer type and fertilization method, depth, and amount per year.

Treatments	Types of N fertilizer	N application amount (kg ha^-1^ y^-1^)	N fertilization depth (cm)
CK	Compound fertilizer with N 22%	262.5	0
R0	15% U + 85% CRU	250	0
R6	15% U + 85% CRU	250	6
R12	15% U + 85% CRU	250	12
R6B	15% U + 85% CRU	250	6
R6R	15% U + 85% CRU	200	6

U, common urea; CRU, controlled-release urea. The percentage of N fertilizer species indicates the proportion of N provided by the corresponding fertilizer to the total N applied. Depths of N fertilization refer only to CRU, and common urea was applied as the base fertilizer. The fertilization method refers to that CRU treatment needing a one-time base application, and the CK was applied 6 top dressings.

In January 2022, healthy one-year-old *P. notoginseng* seedlings of uniform size were selected and transplanted into the designated experimental field. Afterward, the plants were allowed to grow for two years before harvest (the first year of growth in the field was referred to as the two years old *P. notoginseng*, and the second year was called the three years old *P. notoginseng*). During the two-year growth period, soil and plant samples were collected to analyze relevant parameters. The annual N input was 250 kg ha^−1^ for R0, R6, R12, R6B, and except for R6R, which received 200 kg ha^−1^. The N fertilizers used were common urea (46% N) (Hunan Xiangnong Agricultural Group Co., Ltd.) and CRU (with 44% N and 2% polyurethane used for coating) (Ningxia Ronghe Green Technology Co., Ltd.). The CRU used was made of polyurethane material wrapped with urea, ranged in size from 2.5 to 4.5 mm and had a release period of 180 days. At 25°C, approximately 20%-30% of the total N is released within the first 30 days, 40%-50% within 120 days, and the remaining N is completely released by 180 days. When calculating the application rate of CRU, only urea portion (44% N) in the CRU was considered as the N source. The polyurethane coating controls the release of this N over 180 days but does not contribute to the total N available to the plant. Phosphorus (calcium superphosphate, P_2_O_5_ 16%) and potassium (potassium sulfate, K_2_O 52%) fertilizers were applied to a soil depth of 6 cm with the annual rates of 225 kg ha^−1^ (P_2_O_5_) and 300 kg ha^−1^ (K_2_O) (Ou et al., 2020a), respectively, for all treatment except for the CK. The biochar, derived from bamboo, contains 58.92% carbon (C) and 0.53% N, was applied once at 7500 kg ha^-1^ to the two years old *P. notoginseng* in R6B.

In CK and R0 treatments, all fertilizers, including N, P, and K, were applied to the soil surface. In 2022, fertilizers for R6, R6B, and R6R were placed at the bottom of planting holes with a depth of 6 cm and mixed with small amount of surrounding soil. In R12 treatment, N fertilizer was positioned at the base of the planting holes, reaching a depth of 12 cm. Before P and K fertilizers were applied, soil was added to adjust the planting holes depth to 6 cm, ensuring consistency with other treatments. Following the fertilization process, the seedlings were placed to a depth of 5–6 cm in the soil. By fixing the ridge width and planting rows, and using a specialized hoe to create planting holes, consistent fertilization distance was maintained. In each CRU fertilization treatment (R0, R6, R12, R6B, and R6R), CRU and common urea accounted for 85% and 15%, respectively to ensure sufficient N availability during the seedling stage (**Table 1**). The CK treatment involved conventional fertilization, with six surface topdressings from May to October each year after transplantation at a fertilization rate of 239 kg ha^−1^ (22-7-11 of compound fertilizers), resulting in the total input of 262.5 kg ha^−1^ N, 285 kg ha^−1^ P_2_O_5_, and 180 kg ha^−1^ K_2_O.

During the growth of three years old *P. notoginseng* (i.e., the second year of the field trial), a top dressing was applied once in May for all CRU treatments, and the fertilization amounts and depths consistent with those of the two years old *P. notoginseng* (i.e., the first year of the field trial). In R0, all fertilizers (N, P and K) were applied to the soil surface. For R6, R6B, and R6R, a 6 cm (approximately 2 cm in diameter) deep hole was made along the base of the *P. notoginseng* stem with a Polyvinylchloride (PVC) plastic pipe, all the fertilizer were placed into the hole along the PVC pipe, and then the soil was backfilled. For R12, N fertilizer was applied through PVC pipes to a depth of 12 cm, and the soil was backfilled to a depth of 6 cm before P and K fertilizers were applied.

Across all treatments, 4500 kg of ha^−1^ organic fertilizer was applied before transplanting and the crop density was 375,000 plants ha^−1^. Crop management strategies, including irrigation, weeding, and pest and disease control, were conducted in accordance with local cultivation guidelines (Tuo et al., 2023).

### 2.3 Samples collection and determination

#### 2.3.1 Yield, active components, and heavy metals

##### 2.3.1.1 Yield

An independent subplot with an area of 1 m^2^ was established in each plot to examine seedling emergence, survival rate, and yield. In November, samples were collected from each plot from plants that were two and three years old, and then divided into aboveground and underground components. All plants were cleaned and dried until a consistent weight was achieved at 65°C, with the dry weight recorded. Finally, the yield of *P. notoginseng* was calculated using the biomass (dry weight) of the underground parts (i.e., the roots) of the three-year-old plants.

##### 2.3.1.2 Content of active components and heavy metals

Six plant roots were selected from each plot for drying and grinding to create composite samples. These composite samples were used to detect the content of saponins, total flavonoid, and heavy metals in the roots to evaluate the quality of *P. notoginseng*. Saponins (including ginsenoside Rg1, ginsenoside Rb1, Panax ginsenoside R1, ginsenoside Re, and ginsenoside Rd) and total flavonoid represent the primary and secondary active ingredients of *P. notoginseng*, respectively, to assess the effectiveness of the medicinal material. Heavy metal content (including Cd, As, Pb, Hg, and Cu) was used to evaluate the safety of the medicinal materials. Finally, higher saponins and total flavonoid content, as well as lower heavy metal content, were considered better quality of *P. notoginseng*.

Rg1, Rb1, Rd, Re, and R1 concentrations were measured using high-performance liquid chromatography following the method specified by the Pharmacopoeia of the People’s Republic of China (Part I, 2020) (Pharmacopoeia of People’s Republic of China, 2020). The standard Rg1, Rb1, Rd, Re, and R1 samples were sourced from Suzhou Gris Biotechnology Co., Ltd. The determination was conducted using a kit column measuring 250 mm × 4.6 mm with a particulate size of 5 μm. The mobile phase included a combination of acetonitrile (ACN) and water, with an elution time of 0-20 min. 20% ACN; 22–45 min, 20–22% ACN; 45–50 min, 22–46% ACN; 50–60 min, 46–55% ACN; 60–65 min, 55–20% ACN; and 65–70 min, 20% ACN. The flow rate was maintained at 1.0 mL min^−1^, the injection volume was set at 10 μL, the monitoring wavelength was established at 203 nm, and the thermal level of the column was maintained at 25°C (room temperature) (Li et al., 2023). Total saponins are obtained by summing Rg1, Rb1, Re, Rd, and R1.

The total flavonoid content was determined by spectrophotometry (Matić et al., 2017). Quercetin standard was purchased from Suzhou Gris Biotechnology Co., Ltd. And 0.4 g sample of root powder was measured and combined with 8 mL of a 70% methanol solution. The mixture was agitated for 10 min and subjected to three rounds of sonication (15 min each), followed by centrifugation at 3,500 r min^-1^ for 15 min. At a wavelength of 256 nm, the absorbance of the supernatant was observed, then the total flavonoid content was calculated using a standard curve obtained from the standard absorbance readings.

As and Hg contents in the roots were determined by atomic fluorescence (Zhu et al., 2017), whereas the Pb, Cd, and Cu contents were examined by atomic absorption spectrophotometry in a graphite furnace (Xia et al., 2018).

#### 2.3.2 Plant growth and nutrient uptake

At harvest three plants were randomly chosen from each treatment. The underground section was carefully cleaned and then scanned with a root scanner (Epson V700) to capture images of the roots. Subsequently, the total root length (RL), total root surface area (RSA), and total root volume (RV) were assessed using WinRhizoPro software.

Five plants were gathered from each plot in May, July, and September 2022 and at harvest in 2023 to measure the biomass and nutrient content. These plants were divided into aboveground and underground parts, washed, dried to consistent weight at 65°C, and their biomass (dry weight) was recorded. After grinding and sieving, the concentrations of N, P, and K were analyzed using Kjeldahl digestion, automated colorimetry, and flame photometric analysis, respectively (Peng et al., 2012). Nutrient uptake was calculated by multiplying the biomass by the respective total N, P, and K content. The productivity of partial factors (PFP) of the N fertilizer was assessed by dividing the yield by the respective N input.

#### 2.3.3 Enzyme activity and root vitality

Five plants were chosen from each plot in July 2023. After the veins were removed, samples were obtained from the central part of the leaves. The leaf samples were quickly frozen (-80°C) for preservation until further use for determining glutamine synthetase (GS), NADH-dependent glutamate dehydrogenase (NADH-GDH), and nitrate reductase (NR) activities. The detection method follows the guidelines provided by the manufacturer (Suzhou Grace Biotechnology Co., Ltd.).

In May, July, and September 2022, and July 2023, five plants were screened for root vitality. The roots were carefully washed, transferred into centrifuge tubes, and stored at -80°C Subsequently, the root vitality was determined by 2,3,5-Triphenyltetrazolium chloride (TTC) reduction analysis (Ma et al., 2019).

#### 2.3.4 Soil available nutrients

Samples of soil were taken from a depth of 0–20 cm during May, June, July, September, and October 2022, and November 2023. Soil AP was examined using the 0.5 mol L^−1^ NaHCO_3_ extraction method and molybdenum blue colorimetric analysis, while soil AK was examined using the NH_4_OAc extraction method and flame photometry (Lu et al., 2017).

#### 2.3.5 Spatial distribution of NH_4_^+^-N, NO_3_^−^-N, and roots

In May, July, and September 2022, and July 2023, soil samples were gathered from different layers of each plot (**Supplementary Figure S1**) through horizontal (0–9 cm) and vertical (0–20 cm) sampling of the three-dimensional soil body. Vertically, the soil (0–20 cm depth) was cut every 4 cm, and soil samples were collected horizontally within each layer at 3 × 3 cm^2^ intervals, resulting in 3 × 3 × 4 (36 cm^3^) cubic soil blocks. Soil samples from each plot comprised two diagonally obtained samples, and 45 fresh soil samples were collected. Soil NO_3_^−^-N and NH_4_^+^-N were leached with 1 mol L^−1^ KCl, and their contents were evaluated by a continuous flow injection analyzer (AA3, SEAL, Mequon, WI, USA). Roots were picked from different soil layers with forceps, and their lengths were measured to calculate the root length density (RLD).

### 2.4 Data handling and analysis

Microsoft Excel 2019 and Origin 2021 software were utilized for data analysis and visualization, respectively. RStudio was employed for redundancy and correlation analyses to explore the determinants of yield and total saponin content. One-way ANOVA was conducted using IBM SPSS 22.0 to identify significant differences between treatments. Structural equation modeling (SEM) was performed using Amos 24.0 software (IBM, SPSS, USA) to assess the indirect and direct effects of N-related enzyme activity, spatial-temporal distribution of NO_3_^−^-N and root characteristics on yield. The model’s fit to the data was assessed through the chi-square (χ^2^, 0.05 < P ≤ 1.00) test, index of goodness-of-fit (0.95 < GFI ≤ 1.00), and the root mean square approximation error (0 ≤ RMSEA ≤ 0.05) (Livote and Wyka, 2009).

## 3 Results

### 3.1 Yield, active components, and heavy metal content in P. notoginseng

**Figure 1:**
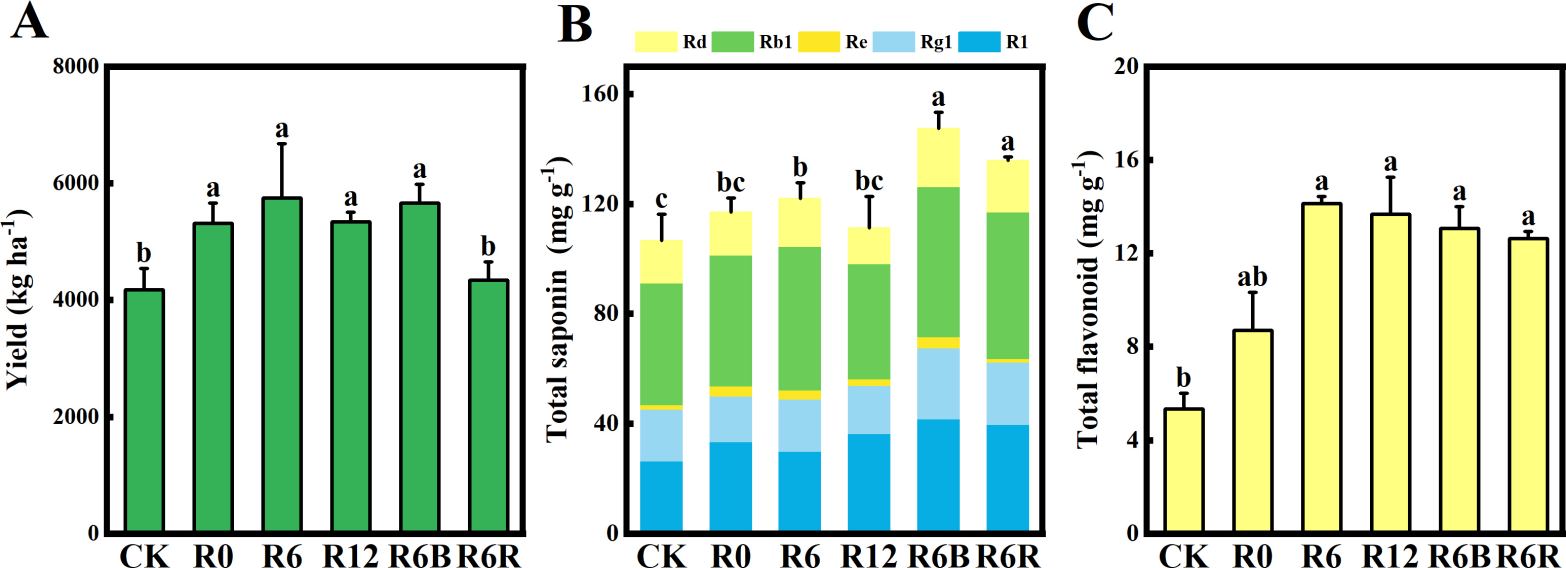
Yield **(A)**, total saponin **(B)**, and total flavonoid **(C)** content of *P. notoginseng* under varying treatments. The labels above the columns signify statistically marked differences between the treatments (*P* < 0.05). R1, notoginsenoside R1; Rg1, ginsenoside Rg1; Re, ginsenoside Re; Rb1, ginsenoside Rb1; Rd, ginsenoside Rd.

At the same N application rate, the yields of R0, R6, and R12 increased by 27.1%, 37.6%, and 27.9% compared with CK (**Figure 1A**). However, there were no significant variations between the three application depths. In comparison to CK, the yield increased by 27.9% with R6B, and was maintained with R6R, despite a 20% reduction in N input. This is consistent with the hypothesized results. In addition, no notable difference was observed in the emergence rate among the treatments, whereas the survival rate of R6R was markedly lower compared to CK (**Supplementary Table S1**).

The total saponin contents of R6B and R6R were substantially higher compared to the other treatments (**Figure 1B**). Under the same N application rate (R0, R6, and R12), no substantial difference was observed in the total saponin content among the distinct fertilization depth treatments. Compared with CK, the differences in R1, Rg1, and Rb1 among the different CRU fertilization depths were not significant, but the Re content increased significantly (**Supplementary Figure S2A**). The Re content of CRU at different fertilization depths (R0, R6, and R12) decreased with increasing fertilization depth. Additionally, the Rd of the R12 treatment was markedly reduced compared to the other treatments. Except for R0, the total flavonoid content of R6R, R6B, R12, and R6 increased significantly by 1.37–1.65 times compared to CK (**Figure 1C**).

The contents of Cd, As, Pb, Cu, and Hg in the roots in all treatments were below the limit values of “Green guidelines for medicinal plants and products in international commerce” (**Supplementary Figure S2B**). Compared with CK, the contents of As, Pb, Cu, and Hg showed no change under different CRU fertilization depths, but the levels of Cd, As, Pb, Cu, and Hg in roots were reduced in R6B. Notably, the Cd, As, Pb, Cu, and Hg contents of R6R were higher than those of CK.

### 3.2 Temporal and spatial distribution of NO₃⁻-N in soil

**Figure 2:**
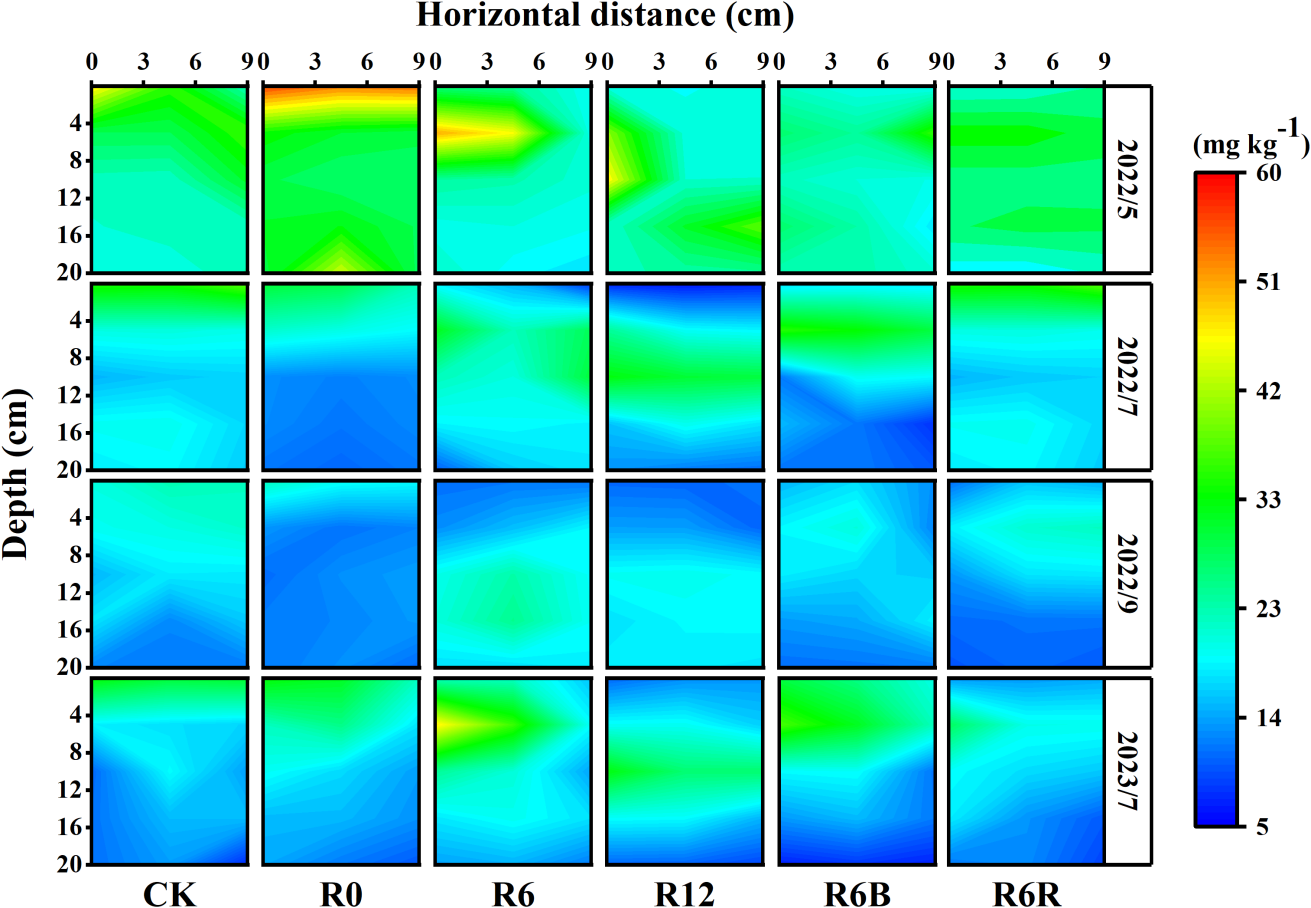
Spatial distribution of soil NO_3_^−^-N under varying treatments during four time periods in May, July, September 2022, and July 2023. The horizontal extent was measured at the base of the *P. notoginseng* stem. At the same time, the vertical distance (depth) denotes the depth measured vertically from the uppermost layer of soil, taken as the zero point. 2022 corresponded to the two years old while 2023 corresponded to the three years old of *P. notoginseng*.

Compared to surface application treatments (CK, R0), the soil NO_3_^−^-N distribution was more concentrated in deep application treatments (R6, R12, R6B, R6R), forming a distribution hot zone (**Figure 2**). In CK, soil NO_3_^−^-N was concentrated within 0–8 cm depth, and the content declined swiftly with the enhance in soil depth. With the increase of fertilization depth, an increase in the depth of the NO₃⁻-N hot zone (concentration > 20 mg kg⁻¹) was observed in deep CRU treatment.

Vertically, the NO_3_^−^-N in R0, R6, and R12 were mainly distributed in 0–2 cm, 4–8 cm, and 8–16 cm in May 2022. In July 2022, the NO_3_^−^-N content decreased compared with that in May 2022, but the NO_3_^−^-N distribution hot zone remained concentrated in the fertilization area. However, only deep application treatments (R6, R12, R6B, R6R) maintained high NO_3_^−^-N concentrations (concentration > 15 mg kg^−1^) in the root zone in September 2022. After fertilization in 2023, the distribution of soil NO_3_^−^-N was similar to that in 2022. In the horizontal direction, the deep application of CRU treatment had a widely distributed NO_3_^−^-N hot zone (0–9 cm). Although there was a significant reduction in soil NO_3_^−^-N concentration following N application, the NO_3_^−^-N hotspot (concentration > 20 mg kg−1) for R6R continued to exist in the soil layer 0–8 cm deep.

The NH_4_^+^-N distribution hot zone in May and July were similar to those of NO_3_^−^-N, and the content gradually decreased over time, reaching a lower level in September (**Supplementary Figure S3**). Additionally, compared to R6, adding biochar at a depth of fertilizer application of 6 cm reduced the NH_4_^+^-N content in the soil, and the average NH_4_^+^-N in the 0–12 cm soil layer in R6B was lower than that in R6.

### 3.3 Root characteristics of *P. notoginseng*

**Figure 3:**
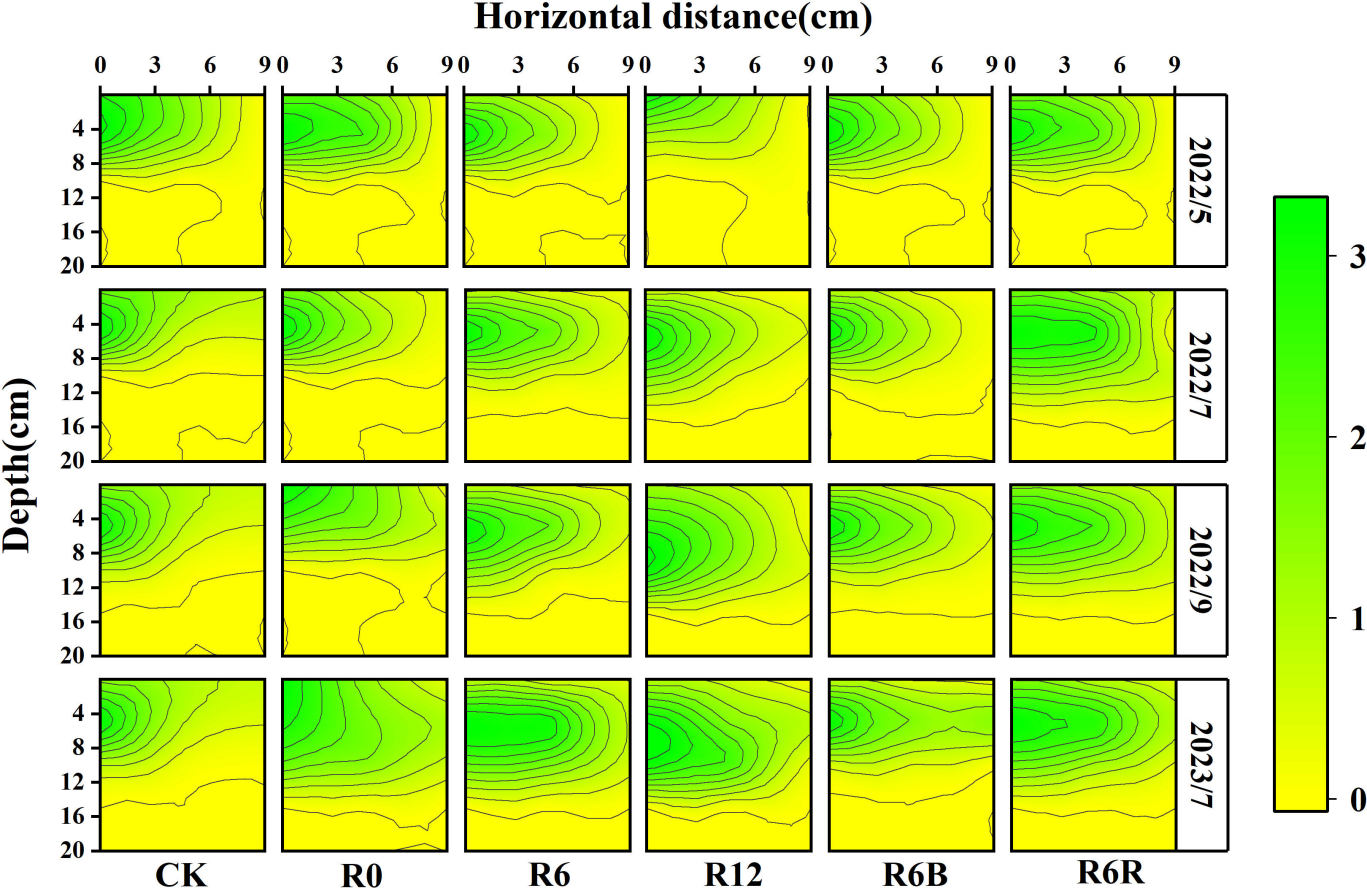
Spatial distribution of roots under different fertilization treatments during four time periods in May, July, September 2022, and July 2023. The horizontal extent was measured at the base of the *P. notoginseng* stem. At the same time, the vertical distance (depth) denotes the depth measured vertically from the uppermost layer of soil, taken as the zero point. The year 2022 corresponded to the two years old while 2023 referred to the three years old of *P. notoginseng*.

As time progressed and the depth of CRU placement increased, the RLD distribution range expanded laterally (0–9 cm) and extended deeper (0–16 cm), with the core root distribution zone (RLD ≥ 2 cm) gradually shifting downward (**Figure 3**). Meanwhile, R6R resulted in a larger distribution range and depth than CK. At a fertilization depth of 6 cm, the RLD was observed in the order of R6B > R6 > R6R.

Under an identical N application rate, only the 6 cm fertilization depth treatments (R6 and R6B) increased the root characteristics (RL, RSA, and RV) of three years old *P. notoginseng* (**Supplementary Table S2**) compared to CK. The root characteristics showed no significant differences between R6 and R6B. However, the RL, RSA, and RV in R6R decreased significantly by 30.3%, 26.2%, and 31.9%, respectively, compared with R6. No substantial differences in root characteristics were detected between R12 and CK.

Throughout the growth period, R6R consistently maintained lower root vitality, whereas R6B exhibited higher root vitality among all the treatments (**Supplementary Figure S4**). In contrast, the root vitality of R12 was initially higher but declined later in the growth period. Notably, the root activity in R6 and R6B remained high during the late growth stages (September 2022).

### 3.4 Nutrient uptake of N, P, and K in *P. notoginseng*

In most instances, R6B consistently exhibited higher levels of soil AP and AK than the other treatments (**Supplementary Tables S3** and **Supplementary Table S4**). Except for a decrease in P and K content at harvest (November 2023), the underground N, P, and K contents remained lower than those in the aboveground parts throughout most of the growth period, with the P content reaching its highest level in September (**Supplementary Figure S5**).

**Figure 4:**
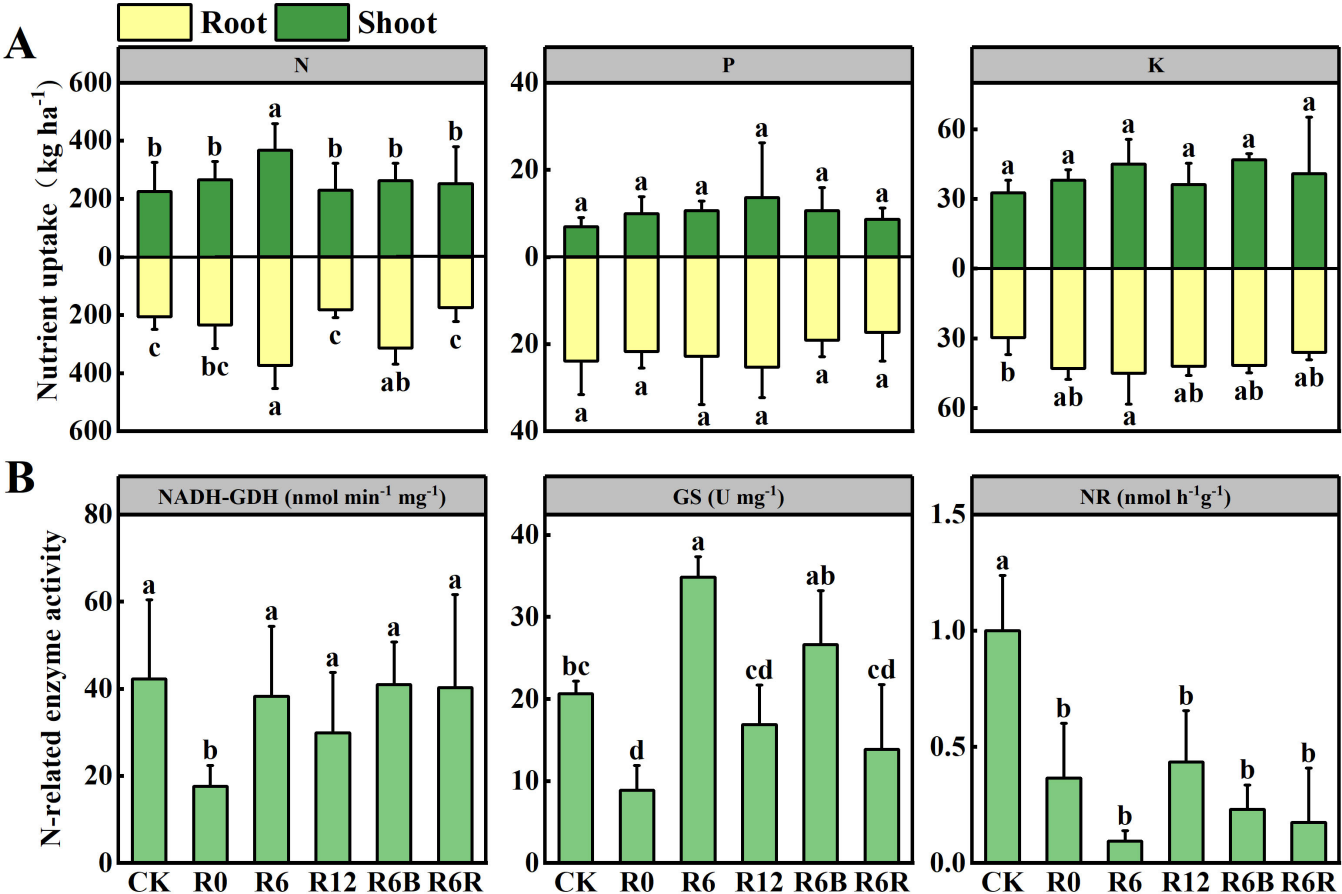
Nutrient uptake **(A)** of N, P, and K in the aboveground and underground parts at harvest under different fertilization treatments. N-related enzyme activity **(B)** under different fertilization treatments. NR, Nitrate Reductase; GS, Glutamine Synthetase; NADH-GDH, NADH-dependent Glutamate Dehydrogenase. Data with different letters represent statistically significant (*P* < 0.05).

Compared to CK, GS activity increased at the 6 cm fertilization depth (R6 and R6B), while NR activity decreased (**Figure 4B**). Except for R0, there was no marked differences in NADH-GDH activity between the other treatments and CK.

The total N uptake content gradually declined as fertilization depth increased during the growth period (except in September) for the two years old *P. notoginseng*. No notable differences in P and K uptake were found between the growth periods (**Supplementary Figure S6**). Compared to CK, R6 and R6B increased underground N uptake at harvest. However, only R6 increased both aboveground and total N uptake (**Figure 4A**). There were no substantial differences in P and K acquisition at harvest among treatments, except for the underground K acquisition of R6, which higher than CK.

No notable differences were observed in PFP among R0, R6, R12, and R6B, but they were all higher than those in CK and R6R (**Supplementary Figure S7**).

### 3.5 Factors affecting the yield and quality of *P. notoginseng*

**Figure 5:**
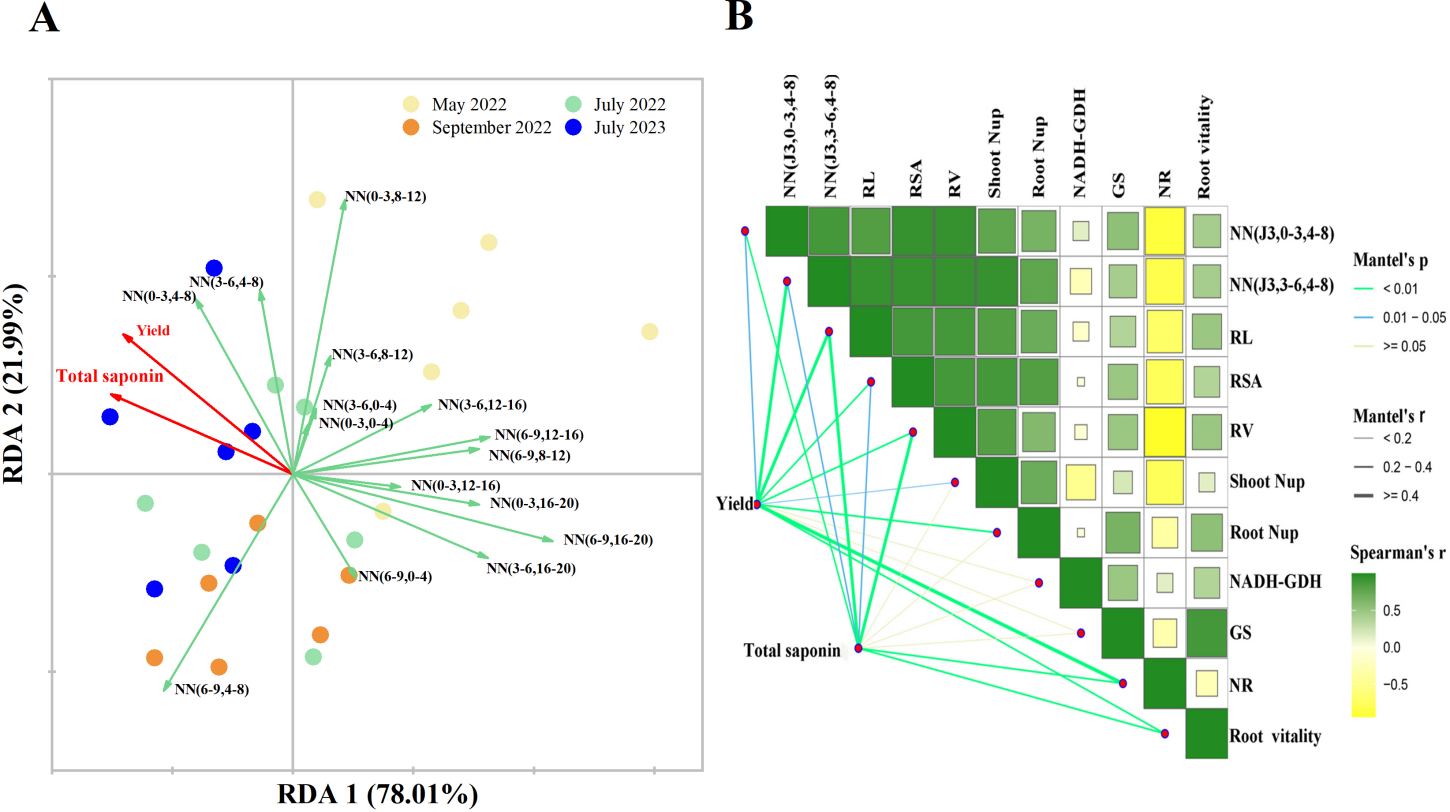
Redundancy analysis **(A)** of soil NO_3_^−^-N, *P. notoginseng* yield and total saponin content at different stages and at different fertilization depths. Correlation analysis **(B)** between soil NO_3_^−^-N and key physiological-biochemical parameters of *P. notoginseng*. NN (a-b, c-d) refers to soil NO_3_^−^-N content in micro areas with a-b cm horizontally and c-d cm, for example NN (0-3, 0-4) meaning soil NO_3_^−^-N concentration in 0-3 cm horizontally and 0-4 cm vertically. In panel **(B)**, the width of each line correlates with the partial Mantel’s r values, while the color of the lines reflects statistical significance, determined by 999 permutations. The gradient in Pearson correlation coefficients represents the correlation. NN (J3,0-3, 4-8), the soil NO_3_^−^-N content of three years old *P. notoginseng* in July was 0-3 cm horizontally and 4-8 cm vertically; NN (J3,3-6, 4-8), the soil NO_3_^−^-N content of three years old *P. notoginseng* in July was 3-6 cm horizontally and 4-8 cm vertically; Root Nup, underground N uptake at harvest time; Shoot Nup, aboveground N uptake at harvest time; RL, root length at harvest time; RSA, root surface area at harvest time; RV at harvest time, root volume; GS, Glutamine synthetase; NR, nitrate reductase; NADH-GDH, NADH-dependent Glutamate Dehydrogenase; Root vitality, the root vitality of three years old *P. notoginseng* in July.

According to RDA, RDA1 and RDA2 explained 78.01% and 21.99% of the variance in yield and total saponin content, respectively (**Figure 5A**). In July 2023 (July of three years old *P. notoginseng*), soil NO_3_^−^-N micro areas (0–3, 4–8, i.e., horizontal 0–3 cm, vertical 4–8 cm) and (3–6, 4–8, i.e., horizontal 3–6 cm, vertical 4–8 cm) were the main factors affecting yield and total saponin content. Further correlation analyses showed that yield and total saponin content were significantly and positively correlated with total RL, RV, NR, and root vitality (P > 0.05) (**Figure 5B**). In particular, total saponin and flavonoid were correlated with plant N content (**Supplementary Figure S8**). In addition, the yield was affected by the soil NO_3_^−^-N in micro area (J3, 3–6, 4–8), and the total saponin was also affected by the soil NO_3_^−^-N in (J3, 0–3, 4–8) micro area. The soil NO_3_^−^-N in (J3, 0–3, 4–8) and (J3, 3–6, 4–8) micro areas showed a strong positive correlation with RL, RSA, and RV, and aboveground and underground N uptake, and negatively correlated with NR.

**Figure 6:**
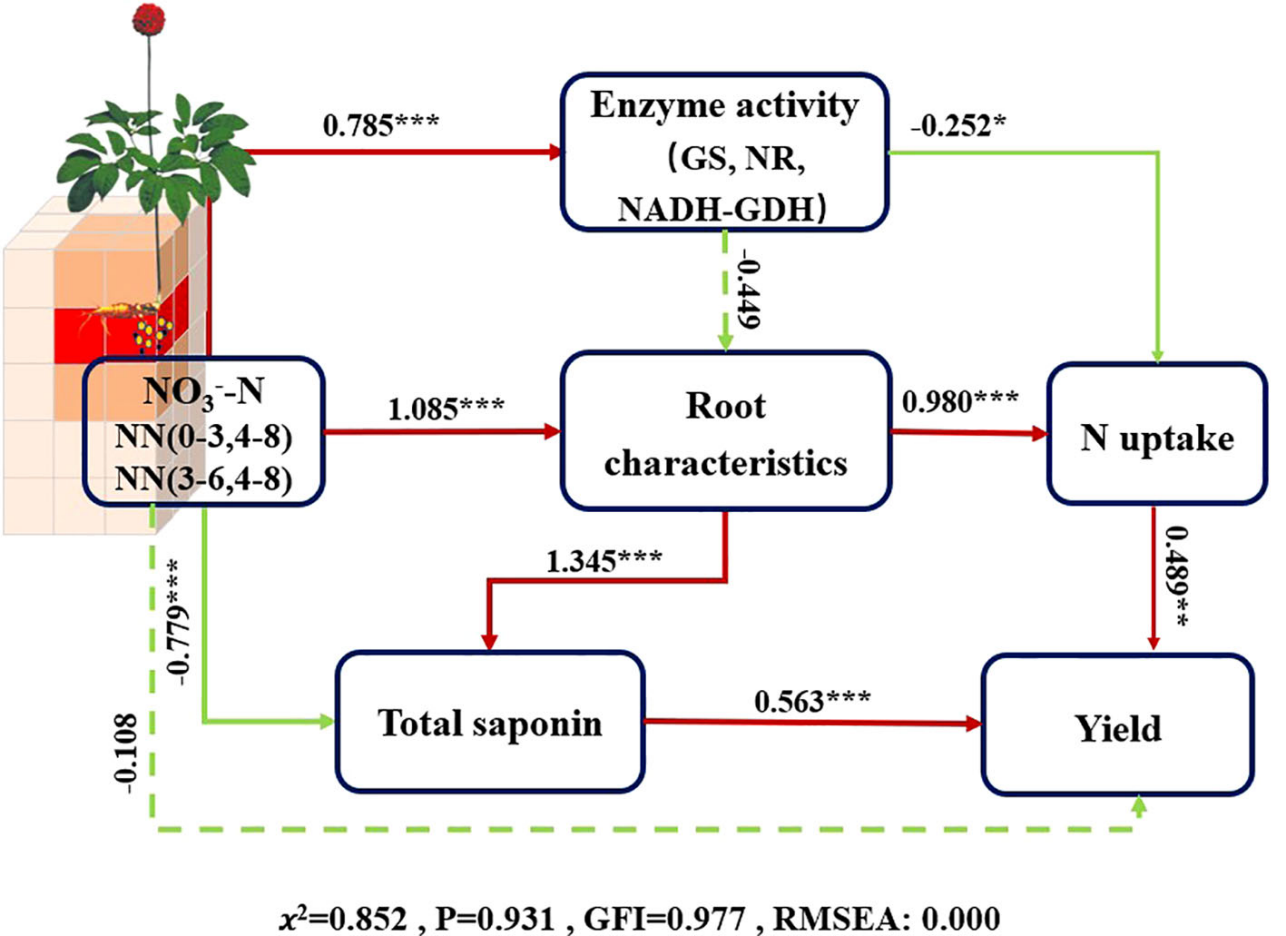
Structural equation model (SEM) illustrating the influence of key factors on the yield and total saponins of *P. notoginseng*. Red lines represent positive correlations, green lines denote negative correlations, and paths with non-significant coefficients are shown as dashed lines. The thickness of the arrow lines reflects the magnitude of the normalized path coefficients, while the numbers on the lines denote the standardized total effects. ***P < 0.001; **P < 0.01; *P < 0.05. NN (a-b, c-d) refers to soil NO_3_^−^-N content in micro areas with a-b cm horizontally and c-d cm, for example NN (0-3, 4-8) meaning soil NO_3_^−^-N concentration in 0-3 cm horizontally and 4-8 cm vertically; Root characteristics, a general term for root length, root surface area, and root volume at harvest time; N uptake, a general term for N uptake in the aboveground and underground parts during the harvest period.

According to the SEM analysis, soil NO_3_^−^-N in (0–3, 4–8) and (3–6, 4–8) microareas exerted a direct negative effect on total saponin content. Soil NO_3_^−^-N in microareas (0–3, 4–8 and 3–6, 4–8) indirectly enhanced yield by directly influencing root characteristics and promoting N uptake (**Figure 6**). Although the high NO_3_^−^-N content in the micro areas was not conducive to total saponin accumulation, it ultimately achieved an increase in total saponin content by affecting root characteristics and decreasing the plant N content.

## 4 Discussion

### 4.1 Effect of deep application of CRU on spatiotemporal distribution of soil NO_3_^−^-N

Compared with, CK and R0, the distributions of soil NO_3_^−^-N in the deep application of CRU (R6, R12, R6B, R6R) moved down and became spatially concentrated (concentration > 20 mg kg^−1^). The application placed at a depth of 6 cm increased its distribution depth while not reducing the NO_3_^−^-N content near the surface (**Figure 2**). This resulted in an increased NO_3_^−^-N accessibility for the roots, thereby stimulating the root growth and N uptake. This finding aligns with the results of an earlier study (Zhang et al., 2000). The anaerobic conditions at the fertilization depth (6 cm) may not be conducive to rapid production and subsequent diffusion of NO_3_^−^-N. With soil depth increases, soil oxygen concentration decline, the water content reduce, and the activity of nitrifying microorganisms decreases, leading to the a weakened soil nitrification (Ke et al., 2018). This curbs excessive NO_3_^−^-N buildup and lowers the risk of leaching, while maintaining a long term base level of NO_3_^−^-N (Rychel et al., 2023). However, in growing season, the soil water in the subsurface layer primarily shifted vertically downward, with weak horizontal migration (Jiao et al., 2017; Liu et al., 2020); as a result, the horizontal distribution range of NO_3_^−^-N narrowed, and finally a distribution hot zone was formed (Dou and Sun, 2024). This phenomenon may be attributed to the combined effects of soil structure, microbial activity, and moisture dynamics.

We observed that soil NH_4_^+^-N remained at a relatively low concentration and rapidly decreased in September, indicating that the release of CRU was nearly complete (**Supplementary Figure S3**). Studies have shown that abundant NH_4_^+^-N inhibited the root growth of *P. notoginseng* but NO_3_^−^-N can alleviate this inhibition effect (Ou et al., 2020b). Therefore, the low concentration of NH_4_^+^-N maintained after the deep application of CRU may be advantageous for the growth and yield improvement of *P. notoginseng*. Biochar has a high adsorption capacity on NH_4_^+^, which improves the soil retention of N nutrients and provides a more sustainable N source for crops (Marcińczyk and Oleszczuk, 2022). In addition, this work showed that R6B consistently maintained an elevated NO_3_^−^-N concentration, possibly related to the biochar application reducing N loss and enhancing nitrification (Huang et al., 2023). This synergistic effect could potentially optimize N use efficiency and soil fertility over the long term. On the contrary, a 20% decrease in N content (R6R) only maintained high NO_3_^−^-N concentration in May and July, with a sharp decrease in NO_3_^−^-N concentration in September, which may lead to a shortage of N supply in the later period (Cun et al., 2023).

### 4.2 Impact of deep application of CRU on N uptake and yield

The RLD distribution shifted downward, and the roots became more elongated as the CRU application depth increased (from 0 to 6 cm to 12 cm) (**Figure 3** and **Supplementary Table S2**). Comparable results have been documented in other studies (Cheng et al., 2020). Plant roots characterize nutrient-induced growth (Wu et al., 2022), so the root distribution moves down with the downward movement of the soil NO_3_^−^-N hot zone. This also indirectly leads to enhanced uptake of water or other nutrients (such as P and K fertilizers) by *P. notoginseng* (**Figure 4A**), which is beneficial for plant growth.

This study demonstrated that root of *P. notoginseng* was concentrated at a vertical depth of 4–8 cm (**Figure 3**). Taking into account the downward migration characteristics of NO_3_^−^-N (Gai et al., 2019), we hypothesized that the optimal N application depth is 6 cm, and the experimental results also confirm this. In R6, the hot zone of NO_3_^−^-N overlapped with the root distribution, but the application depth of 12 cm (R12) resulted in a deeper hot zone of NO_3_^−^-N than its concentrated root distribution depth (**Figures 2**, **3**). Ultimately, a higher overlap between NO_3_^−^-N and root distribution leads to increased N uptake (**Figure 4A**, **Supplementary Figure S6**), which aligns with prior studies (Zhang et al., 2022). In addition, deep application of N fertilizer may reduce the environmental loss of N and improve N use efficiency (Qiang et al., 2020). The main reason for this effect may be that deep fertilization increases the resistance to the diffusion of ammonium ions (NH₄⁺) between the soil, reducing the migration of NH₄⁺ toward the soil surface, while also increasing the proportion of NH₄⁺ adsorbed on the soil particle surfaces, thereby effectively inhibiting NH₃ volatilization (Yao et al., 2018). However these effects still require further confirmation and quantification.

Our results showed that root N uptake positively correlated with yield (**Figure 5B**). N is a key nutrient for plant photosynthesis, and sufficient N ensures the synthesis of aboveground organic matter in the shoot and its storage in the root system (Chen et al., 2018). The main component of the roots of *P. notoginseng* is starch (Ma et al., 2016), so an increase in N uptake in plants effectively promoted the yield. However, the activity of N transformation-related enzymes (GS) was enhanced after deep application of CRU, which promoted N synthesis (Wei et al., 2024); this may also be an important factor in the increase of yield after deep application of CRU. Combined with SEM and RDA, we determined that the deep application of CRU ensures the supply of NO_3_^−^-N at key times (July) and spatial ranges (NN,0–3,4–8) and (NN,3–6,4–8), therefore, as stated in hypothesis 3, the distribution of available N aligned with the distribution of roots, facilitating N uptake, ultimately leading to an increase in yield.

Our work provided key theoretical and technical support for future precision fertilization of *P. notoginseng* cultivation. However, deep application of CRU is time-consuming at the current level of planting technology. Developing supporting machinery and slow-release phosphate and potassium fertilizers are necessary for growers to adopt deep fertilization of CRU on a large scale.

### 4.3 Potential mechanism of CRU to improve quality and yield

Secondary metabolites are the basis for the quality of medicinal plants, which are sensitive to the soil nutrient supply (Alami et al., 2023). Flavonoids and saponins are the main medicinal components and vital SMs produced by *P. notoginseng* in response to stressful environments (Deng et al., 2019). Earlier studies have confirmed that reducing N application can increase the saponin content in *P. notoginseng* (Wei et al., 2020), which may be due to the interaction between N and plant hormones. In this research, the content of total saponins and flavonoids increased by 0.27 and 1.37 times compared with CK after 20% N reduction (R6R). More importantly, by adjusting the form and the method of N fertilization compared with CK, deep application of CRU indirectly reduced the NO_3_^−^-N levels in the root zone at the initial growth stage and maintained the NO_3_^−^-N content in the late growth stage, which may be beneficial for enhancing the SMs of *P. notoginseng* (Zhang et al., 2018).

Our results showed the plant N content was negatively correlated with total saponins, R1, Rg1, and total flavonoids (**Supplementary Figure S8**), which confirmed that N content had key effect on plant SMs in our experimental scenario. These results support the theory of carbon and N balance for the SMs accumulation in plant tissues, that is, the relative influence of C and N metabolism processes (Cui et al., 2019). In addition, we hypothesized that the deep application of CRU alters the N supply intensity, resulting in differences in the expression of functional genes regulating saponin synthesis (Zhang et al., 2020), thereby affecting the quality of *P. notoginseng*, which deserves attention in subsequent studies.

In addition, our research findings are consistent with hypothesis 2, showing that the application of biochar (R6B) significantly increased the contents of Rg1, Rd, R1, and total saponins compared to single CRU application (R6, R12, R6R) and the CK control (**Figure 1B**; **Supplementary Figure S2A**). The C and N input from biochar addition were 4419 kg ha⁻¹ and 40 kg ha⁻¹, respectively. Moderate C input not only promoted the slow release of nutrients and thus enhanced soil fertility but also improved microbial activity in the soil (Zheng et al., 2022). Furthermore, biochar supports the absorption of ions (e.g., NH_4_^+^) (Mia et al., 2017), or the presence of low-molecular-weight active substances (Heaney et al., 2019), which helps reduce ammonia volatilization and may also increase the synthesis of secondary metabolites (SMs). Finally, biochar may stimulate the production of bioactive compounds by reshaped beneficial microbial communities (Kolton et al., 2017). These underlying mechanisms require further research for validation. Notably, biochar application also reduced Cd content in the roots of *P. notoginseng* (**Supplementary Figure S2B**) (Chen et al., 2024). In conclusion, our results indicated that the application of soil amendments, such as biochar, is beneficial for enhancing the quality of *P. notoginseng*. These findings highlight the potential of using biochar as a sustainable agricultural practice, particularly for enhancing the quality and safety of medicinal plants like *P. notoginseng*.

## 5 Conclusion

Compared to conventional fertilization, the one-time application of CRU had a better effect on maintaining the yield and quality of *P. notoginseng*. The suitable fertilization depth (6 cm), the formation of a localized NO_3_^−^-N hot zone, and the direct influence on the root characteristics, promoting N uptake or the total saponin content are the key factors for the yield and quality of *P. notoginseng*. On this basis (CRU applied at a depth of 6 cm), adding biochar is beneficial for increasing the content of saponins and reducing the content of Cd. However, it must be combined with good agronomic practices to ensure a satisfactory emergence rate. Simultaneously, reducing the amount of N fertilizer (R6R) still maintained a yield comparable to conventional fertilization. The analysis indicated that using CRU at a 6 cm depth has promising prospects for the production of *P. notoginseng*.

## Data Availability

The raw data supporting the conclusions of this article will be made available by the authors, without undue reservation.

## Supplementary material

Supplementary Table 1 Emergence rate and survival rate of *P. notoginseng*.

Supplementary Table 2 The total root length, total root surface area, and total root volume of *P. notoginseng* in November.

Supplementary Table 3 Contents of soil available P (mg kg^-1^).

Supplementary Table 4 Contents of soil available K (mg kg^-1^).

Supplementary Figure 1 Three-dimensional coordinate diagram of root system sampling in the field.

Supplementary Figure 2 Saponin content **(A)** and heavy metal content **(B)** of three years old *P.notoginseng* under different fertilization treatments.

Supplementary Figure 3 Spatial distribution of soil NH_4_^+^-N under different fertilization treatments during four time periods in May, July, September 2022, and July 2023.

Supplementary Figure 4 Root vitality of *P. notoginseng* under different fertilization treatments during four time periods in May, July, September 2022, and July 2023.

Supplementary Figure 5 Content of N, P, and K in the above-ground and underground parts of different growth periods *P. notoginseng* under different fertilization treatments.

Supplementary Figure 6 The uptake of N, P, and K nutrients in the above-ground and underground parts of two years old *P. notoginseng* under different fertilization treatments during May, June, July, September, and October.

Supplementary Figure 7 N fertilizer use efficiency under different fertilization treatments.

Supplementary Figure 8 Correlation analysis between N content and secondary metabolites of *P. notoginseng* during the growth period.
