# Supplementary material for: Deep application of controlled-release urea increases the yield and saponin content of Panax notoginseng by regulating soil nitrate distribution
Source: Front Plant Sci. 2025 Jan 23;15:1505702. doi: 10.3389/fpls.2024.1505702 (PMC11799552; doi:10.3389/fpls.2024.1505702)
Supplement: Supplementary Table 1 — Emergence rate and survival rate of P. notoginseng. [file SupplementaryFile1.docx]

**Supplementary Figures and Tables**

**Supplementary Tables**

**Table S1** Emergence rate and survival rate of *P. notoginseng*

| Treatment | Emergence rate (%) | Survival rate (%) |
| --- | --- | --- |
| CK | 0.91±0.02 a | 0.80±0.03 a |
| R0 | 0.91±0.07 a | 0.74±0.05 ab |
| R6 | 0.91±0.05 a | 0.79±0.05 a |
| R12 | 0.90±0.03 a | 0.78±0.04 ab |
| R6B | 0.92±0.04 a | 0.69±0.05 bc |
| R6R | 0.86±0.02 a | 0.62±0.07 c |

Note: CK, conventional N application; R0, 0 cm deep dressing of CRU; R6, 6 cm deep dressing of CRU; R12, 12 cm deep dressing of CRU; R6B, 6 cm deep dressing of CRU and biochar; R6R, 6 cm deep 20% reduction CRU. Data with different letters represent statistically significant (*P* < 0.05).

**Table S2** The total root length, total root surface area, and total root volume of *P. notoginseng* in November

|  | Treatment | Total root length (cm) | Total root surf area (cm^2^) | Total root volume (cm^3^) |
| --- | --- | --- | --- | --- |
| **Two-year-old** | CK | 259.97±64.29c | 87.62±8.55c | 2.35±0.38a |
|  | R0 | 259.97±64.29ab | 99.95±3.20bc | 2.64±0.37a |
|  | R6 | 357.06±17.46b | 99.63±4.50bc | 2.53±0.47a |
|  | R12 | 257.91±13.48c | 98.64±4.51bc | 3.23±0.42a |
|  | R6B | 474.08±95.88a | 129.63±10.20a | 2.86±0.40a |
|  | R6R | 349.71±10.38ab | 104.70±12.28b | 3.27±0.77a |
| **Three-year-old** | CK | 335.18±117.34bc | 145.68±69.90b | 2.69±0.6c |
|  | R0 | 699.30±145.01a | 155.60±27.46b | 3.02±1.20bc |
|  | R6 | 714.77±103.92a | 201.87±65.79a | 5.138±1.80a |
|  | R12 | 414.98±125.89b | 124.36±21.46bc | 2.718±0.41c |
|  | R6B | 824.31±101.09a | 192.95±81.98a | 4.95±1.02ab |
|  | R6R | 497.91±51.22b | 148.98±12.32b | 3.51±0.75bc |

Note: CK, conventional N application; R0, 0 cm deep dressing of CRU; R6, 6 cm deep dressing of CRU; R12, 12 cm deep dressing of CRU; R6B, 6 cm deep dressing of CRU and biochar; R6R, 6 cm deep 20% reduction CRU. Data with different letters represent statistically significant (*P* < 0.05).

**Table S3** Contents of soil available P (mg kg^-1^)

| Treatment | 2022/5 | 2022/6 | 2022/7 | 2022/9 | 2022/10 | 2023/11 |
| --- | --- | --- | --- | --- | --- | --- |
| CK | 20.13±2.07a | 19.79±1.60a | 22.13±0.96ab | 20.80±0.83ab | 22.80±0.47ab | 14.20±0.36cd |
| R0 | 20.61±1.64a | 21.14±1.65a | 21.46±1.68ab | 20.67±1.23ab | 21.22±1.35b | 15.40±1.18bc |
| R6 | 20.60±1.98a | 21.88±2.24a | 21.93±1.17ab | 21.40±1.84ab | 21.25±1.45b | 16.40±0.36b |
| R12 | 21.17±1.58a | 20.09±1.20a | 20.67±2.33b | 21.00±1.68ab | 20.74±1.31b | 13.03±0.21d |
| R6B | 22.15±1.85a | 23.73±1.16a | 24.04±2.31a | 23.48±1.39a | 24.92±1.90a | 24.13±2.29a |
| R6R | 20.62±2.07a | 20.24±2.18a | 20.34±0.14b | 19.97±2.04b | 20.45±1.08b | 14.17±0.59cd |

Note: CK, conventional N application; R0, 0 cm deep dressing of CRU; R6, 6 cm deep dressing of CRU; R12, 12 cm deep dressing of CRU; R6B, 6 cm deep dressing of CRU and biochar; R6R, 6 cm deep 20% reduction CRU. The year 2022 referred to the two years old while 2023 referred to the three years old of *P. notoginseng*. Data with different letters represent statistically significant (*P* < 0.05).

**Table S4** Contents of soil available K (mg kg^-1^)

| Treatment | 2022/5 | 2022/6 | 2022/7 | 2022/9 | 2022/10 | 2023/11 |
| --- | --- | --- | --- | --- | --- | --- |
| CK | 250.67±16.07b | 338.67±14.61a | 346.33±19.66b | 371.33±17.74ab | 366.33±19.14ab | 336.67±10.97b |
| R0 | 367.67±17.47a | 360.50±20.51a | 352.00±13.00b | 355.00±16.37b | 347.67±11.02b | 267.75±7.21c |
| R6 | 376.00±17.78a | 361.67±18.50a | 362.33±17.79ab | 357.67±10.97ab | 356.33±19.86b | 260.75±5.86cd |
| R12 | 365.00±23.64a | 364.33±16.17a | 361.67±16.74ab | 353.33±22.19b | 355.50±19.09b | 293.50±9.02d |
| R6B | 391.33±20.55a | 393.00±6.24a | 386.00±14.42a | 392.00±16.82a | 389.33±14.64a | 372.50±5.51a |
| R6R | 362.67±12.01a | 361.00±16.52a | 358.33±14.29ab | 362.00±9.85ab | 360.33±19.14ab | 284.25±13cd |

Note: CK, conventional N application; R0, 0 cm deep dressing of CRU; R6, 6 cm deep dressing of CRU; R12, 12 cm deep dressing of CRU; R6B, 6 cm deep dressing of CRU and biochar; R6R, 6 cm deep 20% reduction CRU. The year 2022 referred to the two years old while 2023 referred to the three years old of *P. notoginseng*. Data with different letters represent statistically significant (*P* < 0.05).

**Supplementary Figures**


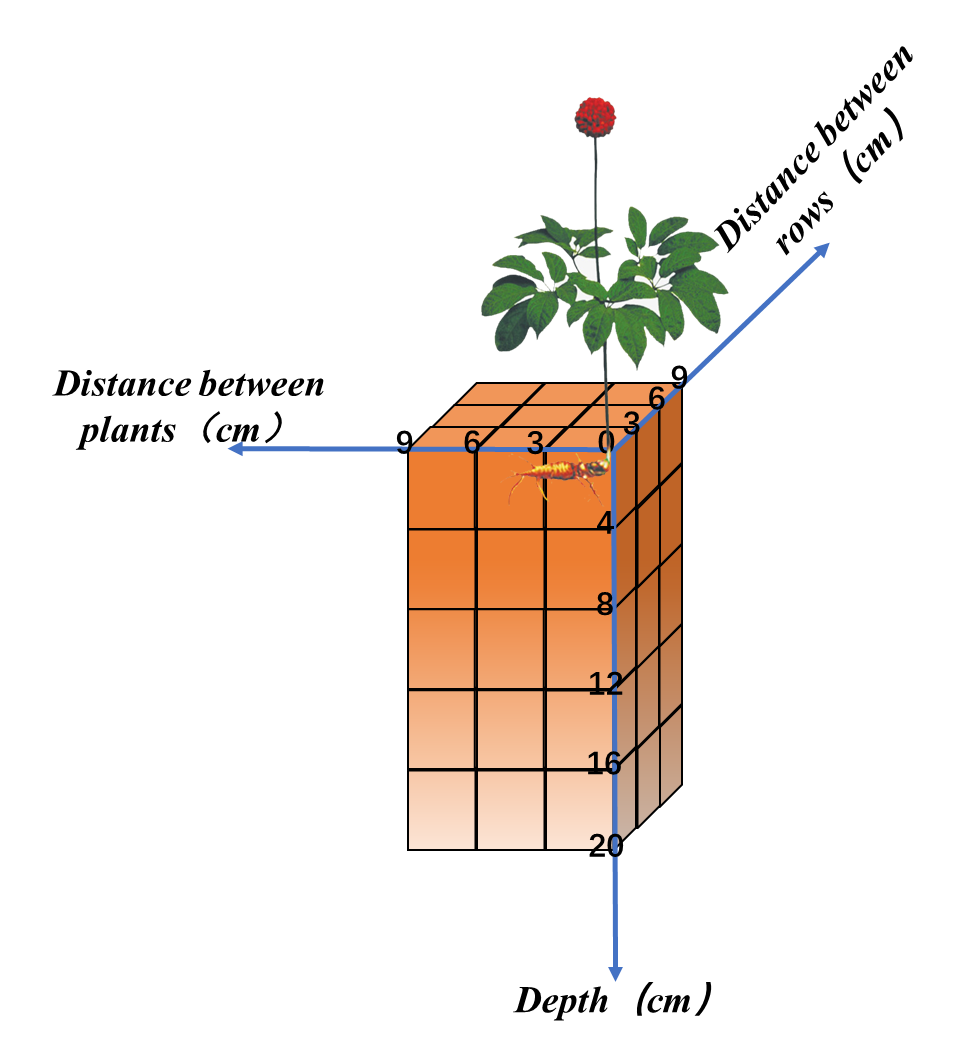


**Fig. S1.** Three-dimensional coordinate diagram of root system sampling in the field. With *P. notoginseng* as the base of the stem as the origin, in both horizontal and vertical directions sampling was carried out in the composed three-dimensional soil mass, where the vertical direction was taken from the three-seven position.


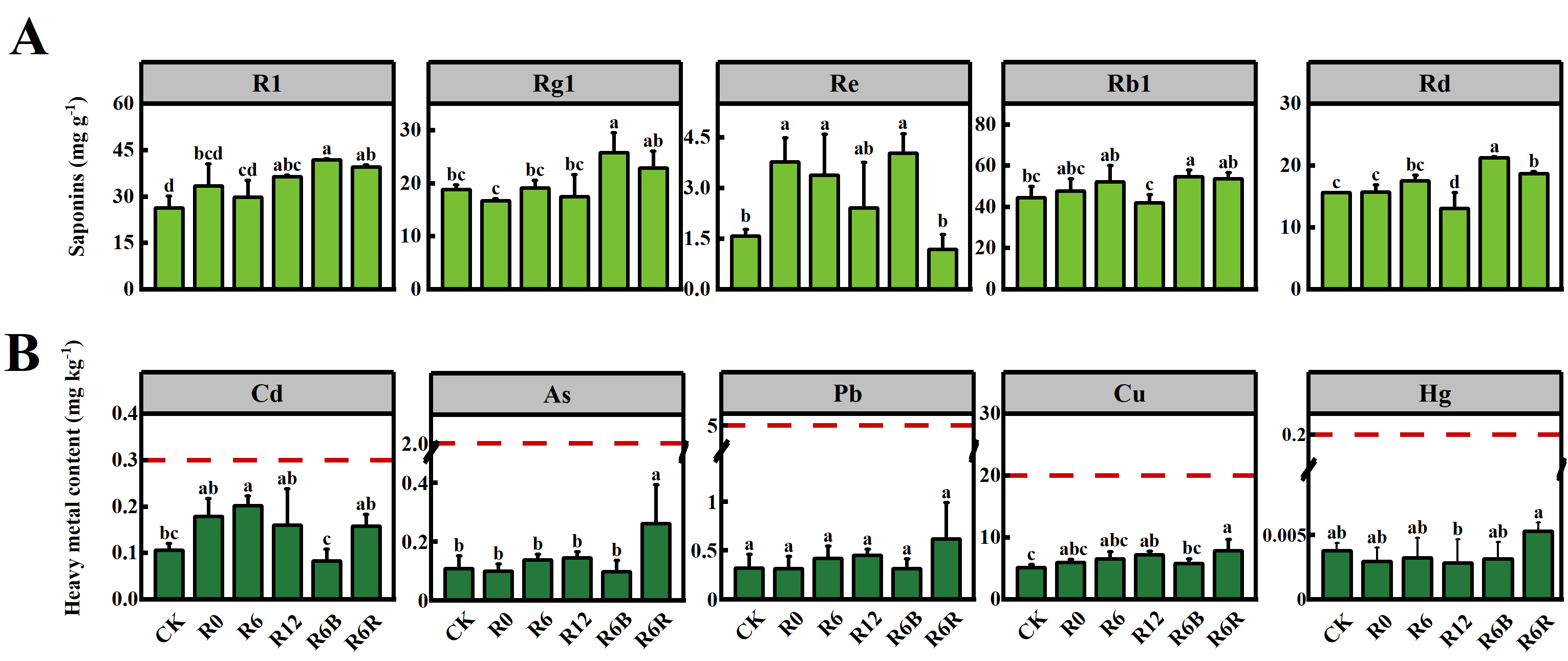


**Fig. S2.** Saponin content **(A)** and heavy metal content **(B)** of three years old *P.notoginseng* under different fertilization treatments. R1, notoginsenoside R1; Rg1, ginsenoside Rg1; Re, ginsenoside Re; Rb1, ginsenoside Rb1; Rd, ginsenoside Rd. The red line in b represent the limit values of heavy metals in “Green standards of medicinal plants and preparations for foreign trade and economy”. Mean ± standard deviation (n = 3). Data with different letters represent statistically significant (P < 0.05).


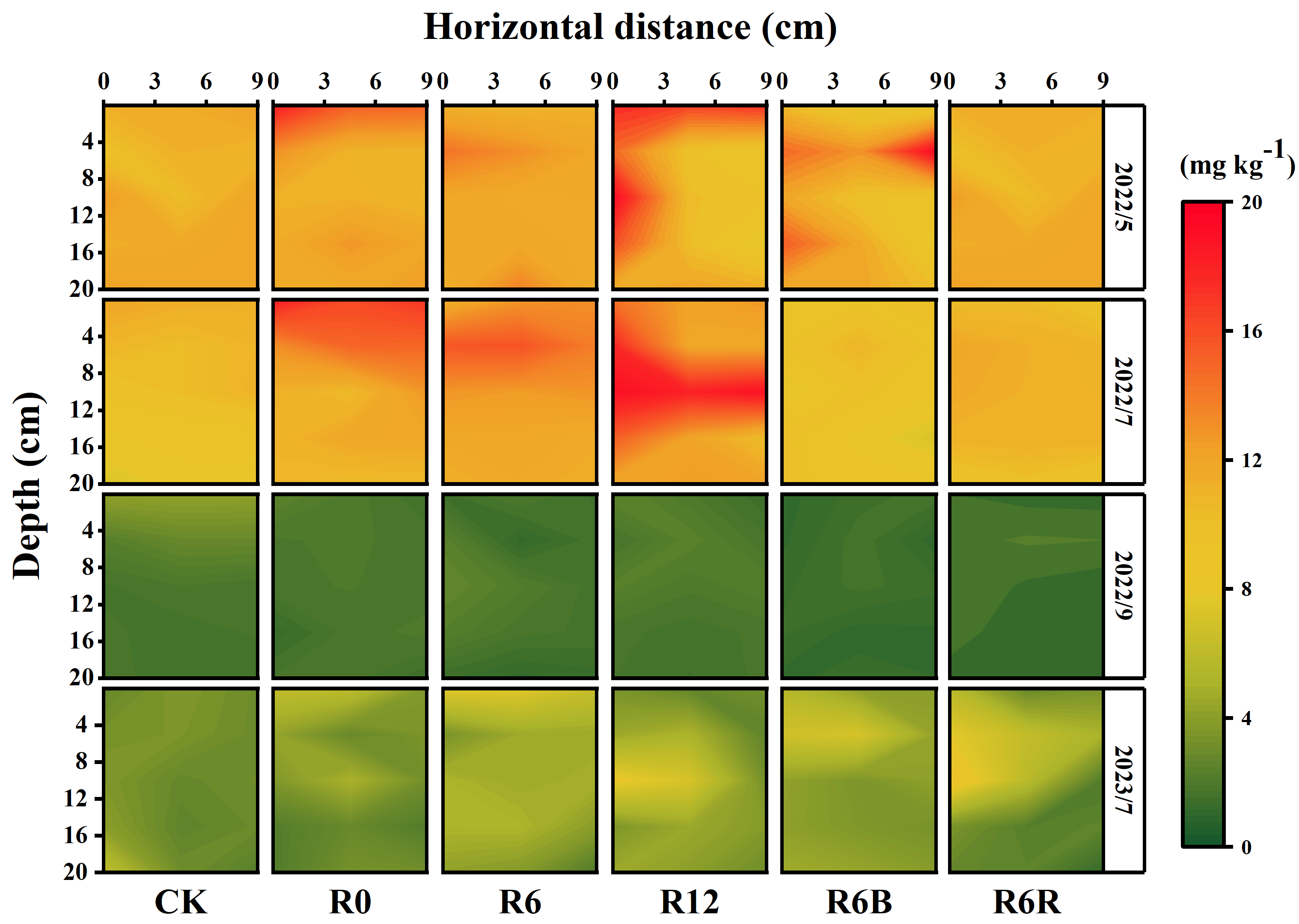


**Fig. S3.** Spatial distribution of soil NH_4_^+^-N under different fertilization treatments during four time periods in May, July, September 2022, and July 2023. The horizontal distance was measured from the base of the *P. notoginseng* stem, while the vertical distance (depth) refers to the vertical depth measured from the soil surface as the zero point. The year 2022 referred to the two years old while 2023 referred to the three years old of *P. notoginseng*.


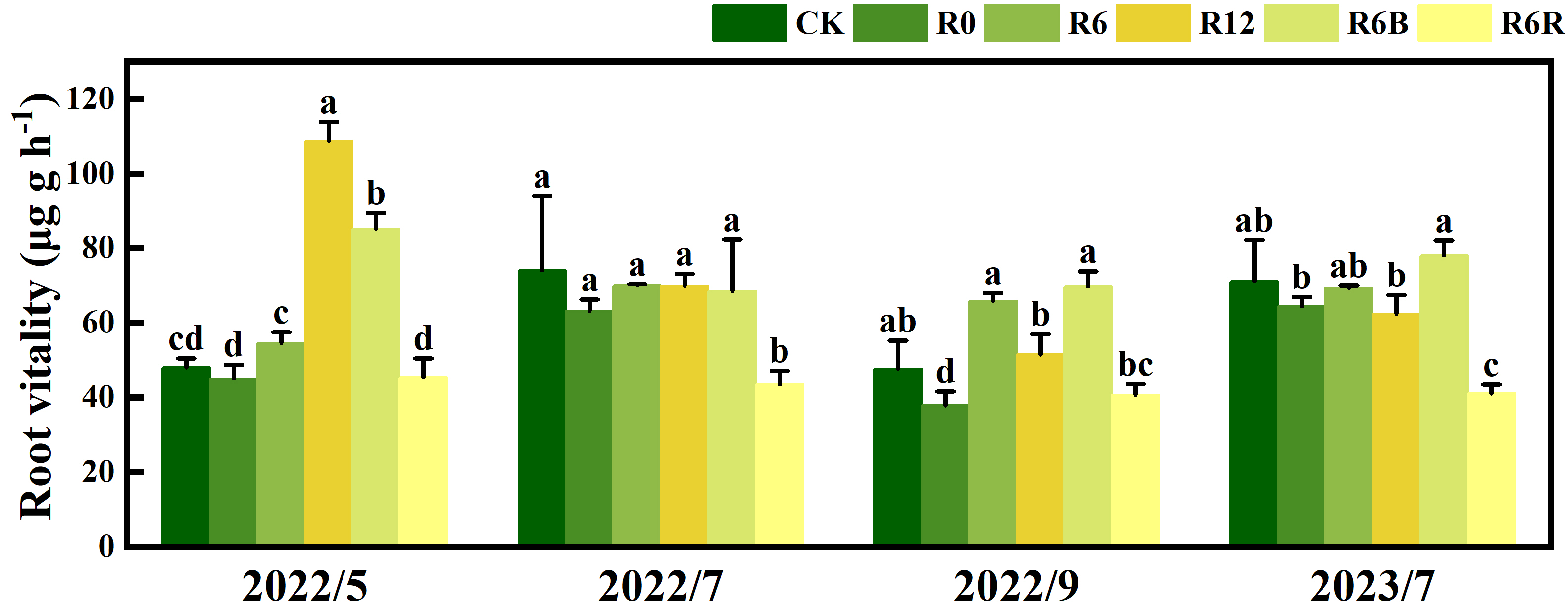


**Fig. S4.** Root vitality of *P. notoginseng* under different fertilization treatments during four time periods in May, July, September 2022, and July 2023. The year 2022 referred to the two years old while 2023 referred to the three years old of *P. notoginseng*. Data with different letters represent statistically significant (*P* < 0.05).


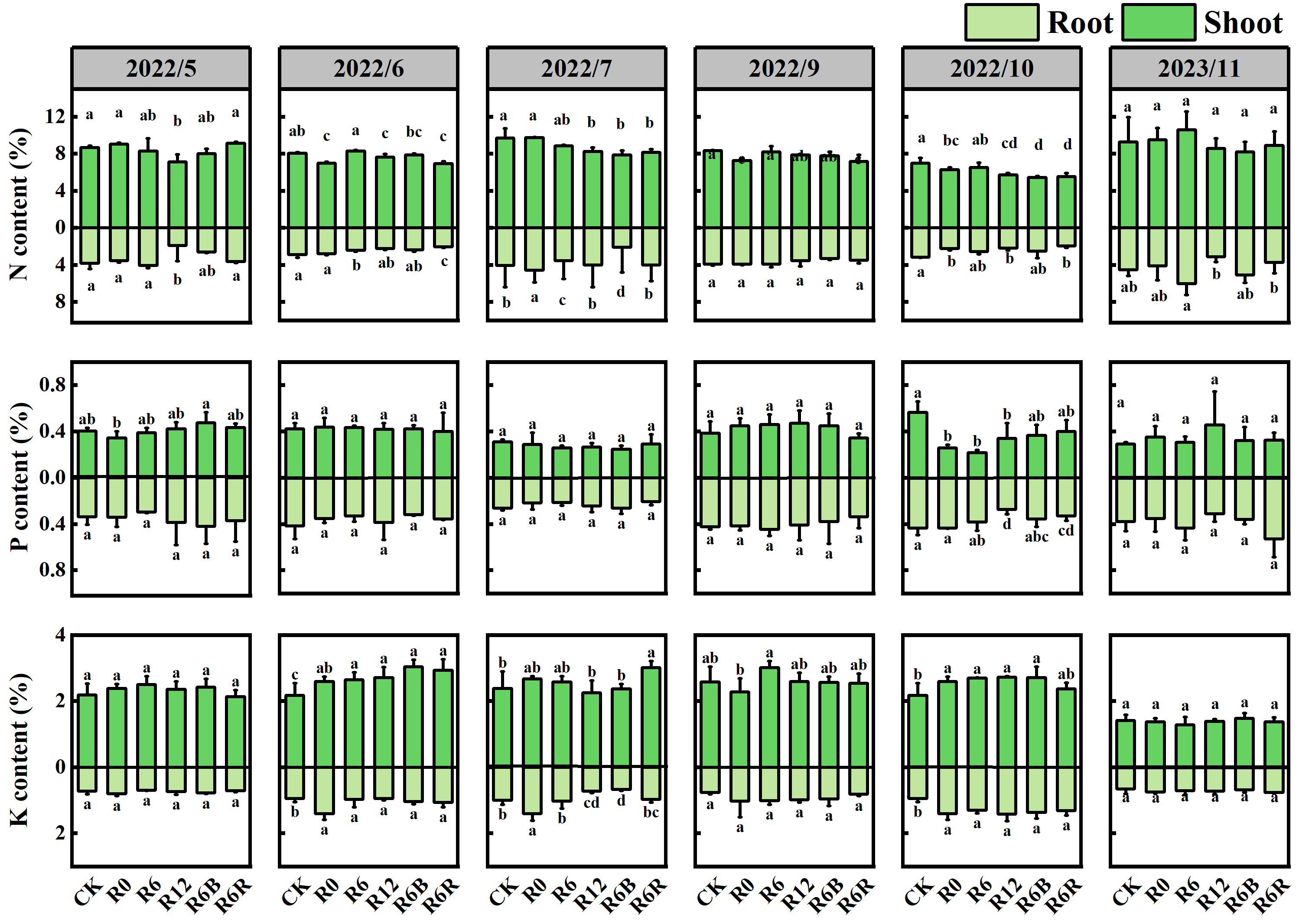


**Fig. S5.** Content of N, P, and K in the above-ground and underground parts of different growth periods *P. notoginseng* under different fertilization treatments. Data with different letters represent statistically significant (*P* < 0.05).


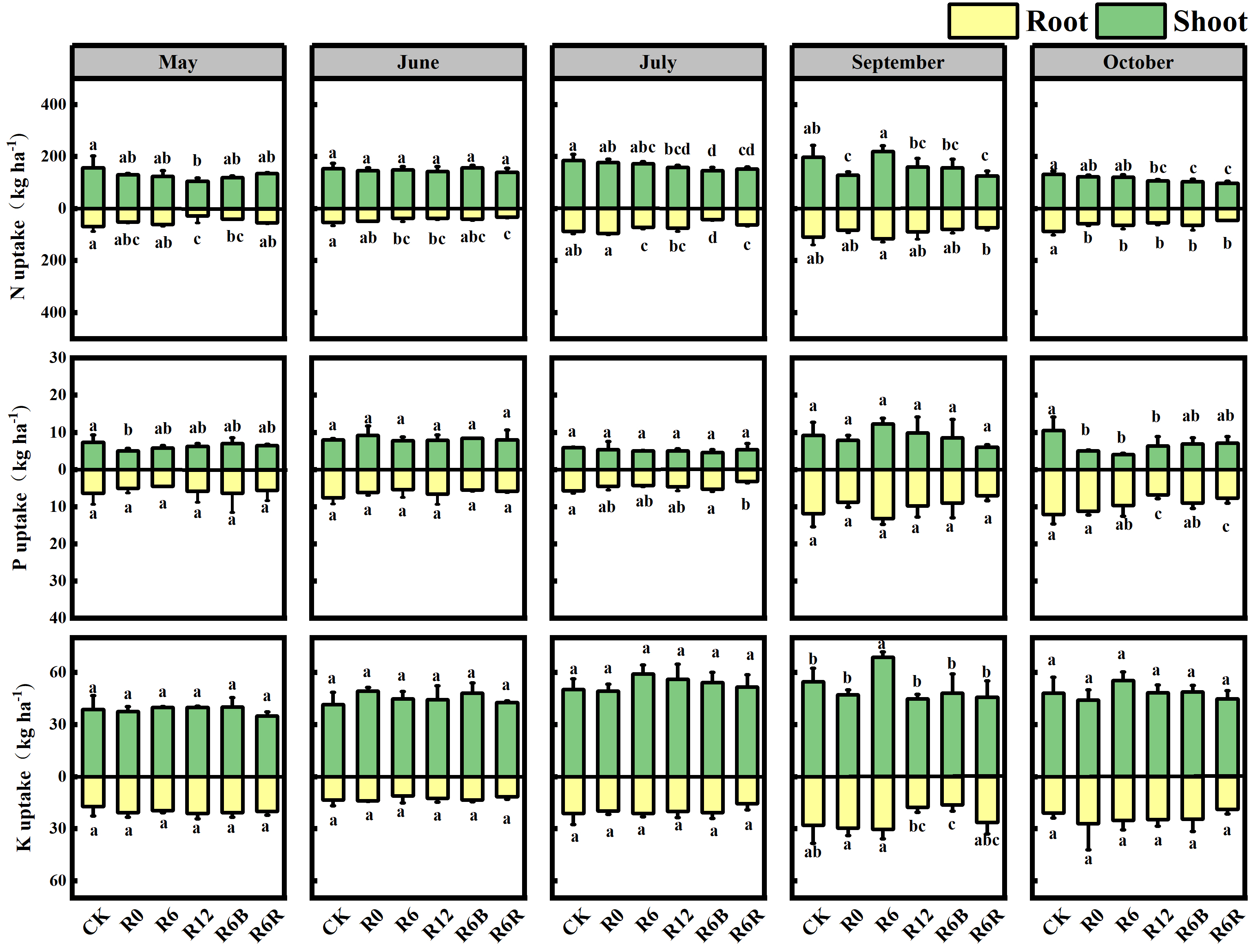


**Fig. S6.** The uptake of N, P, and K nutrients in the above-ground and underground parts of two years old *P. notoginseng* under different fertilization treatments during May, June, July, September, and October. Data with different letters represent statistically significant (*P* < 0.05).





**Fig. S7.** N fertilizer use efficiency under different fertilization treatments. Data with different letters represent statistically significant (*P* < 0.05).





**Fig. S8.** Correlation analysis between N content and secondary metabolites of *P. notoginseng* during the growth period. The correlation is expressed by the color gradient of Pearson correlation coefficient. *, Indicates statistical significance. *, P<0.05, **, P<0.01, ***, P<0.001. R1, notoginsenoside R1; Rg1, ginsenoside Rg1; Re, ginsenoside Re; Rb1, ginsenoside Rb1; Rd, ginsenoside Rd. Data with different letters represent statistically significant (*P* < 0.05).
